# The effect of porous compliance bushings in a dental implant on the distribution of occlusal loads

**DOI:** 10.1038/s41598-024-51429-5

**Published:** 2024-01-18

**Authors:** Katarzyna Młynarek-Żak, Jarosław Żmudzki

**Affiliations:** 1 Department of Engineering Processes Automation and Integrated Manufacturing Systems, Silesian University of Technology, Konarskiego 18a St., 44-100 Gliwice, Poland; 2 Department of Engineering Materials and Biomaterials, Silesian University of Technology, Konarskiego 18a St., 44-100 Gliwice, Poland

**Keywords:** Biomedical engineering, Implants

## Abstract

Porous dental implants are clinically used, but the mechanism of load distribution for stepped implant shaft surrounded by compliance bushings is still not known, especially for different bone conditions. The aim of the study was to assess the impact of the design of a dental implant with compliance bushings (CBs) on the occlusal load distribution during primary and secondary stability using finite element simulation (FEA), with a distinction between low and high quality cervical support under primary stability. The FEA of the oblique occlusal load transfer (250 N; 45°) was carried out for implants under variable bone conditions. The stepped shaft in the intermediate part of the dental implant was surrounded by CBs with an increasing modulus of elasticity of 2, 10 and 50 GPa. With a smaller Young's modulus of the bushings the increase of stress in the trabecular bone indicated that more bone tissue can be protected against disuse. The beneficial effect for the trabecular bone derived from the reduction of the stiffness of the bushings in relation to the loss of the implant's load bearing ability can be assessed using the FEM method.

## Introduction

Biomaterials that mimic the mechanical behavior of bone are developed as a consequence of developing modern technologies of porous materials^1–10^. The porous structure is important for several reasons. One is to create favorable conditions for bone ingrowth^11,12^. This condition can be met by using surface porosity and even by surface treatment and etching^13–17^. The second reason is stress shielding, which results from excessive implant stiffness. The implant bears more load and bone tissue around the implant experiences atrophy from unloading^18–21^. Bulk metals used for load bearing applications demonstrate elastic modulus at least one time larger than the elastic modulus of cortical bone (12–18 GPa) and incomparably larger than cancellous bone (0.1–0.5 GPa)^22,23^. A way to reduce the effect of stress shielding is to reduce the modulus of elasticity^24^, for which purpose technologies of producing porous metal structures are used.

Attempts to use porous metal fabrication technology for load-bearing bone implants have long been known. Sintered materials from metal powders and ceramics as well as metallic foams are historically the oldest. However, they have not been clinically implemented on a large scale due to the problem of achieving a homogeneous open-cell structure as well as pore wall thickness and strength. Increased wall thickness control was achieved thanks to the development of selective laser sintering and melting (SLS, SLM) and electron beam melting (EBM) technologies as well as direct metal printing (DMP) of many biometals, such as titanium, cobalt and alloys and chemical vapor deposition/ infiltration (CVD) of tantalum onto pyrolised polymer foams^8,21,25,26^.

Porous tantalum (PT) has been used clinically in orthopedics^27–29^ and in dentistry^30,31^. Ta shows lower susceptibility to bacterial colonization compared to titanium (Ti)^32^. The surface layer of Ta_2_O_5_ oxide facilitates the deposition of bone-like apatite and accelerates the adherence of osseous tissues^33–36^.

The distribution of loads between the implant and the bone tissue in the case of porous implants is becoming better known and different designs of compliant dental implants are investigated^34,37–43^. However, clinically used implants with porous Ta bushings have been studied only in cooperation with the overdenture^44^.

The mechanism of load distribution for this specific stepped implant shaft surrounded with compliance bushings still not known, especially for different bone conditions. Therefore, it is not known what properties are most desirable in terms of the redistribution of loads in bone tissue and the risk of such an implant being damaged by occlusal forces.

The aim of the study was to assess the impact of the design of a dental implant with compliance bushings on the oblique occlusal load distribution during primary and secondary stability using finite element simulation (FEA), with a distinction between low and high quality cervical support under primary stability. It was hypothesized that the beneficial effects for the trabecular bone derived from the reduction of the stiffness of the bushings surrounding the stepped shaft in the intermediate part of the dental implant in relation to the loss of the implant's load bearing ability can be assessed using the FEM method.

## Material and methods

**Figure 1:**
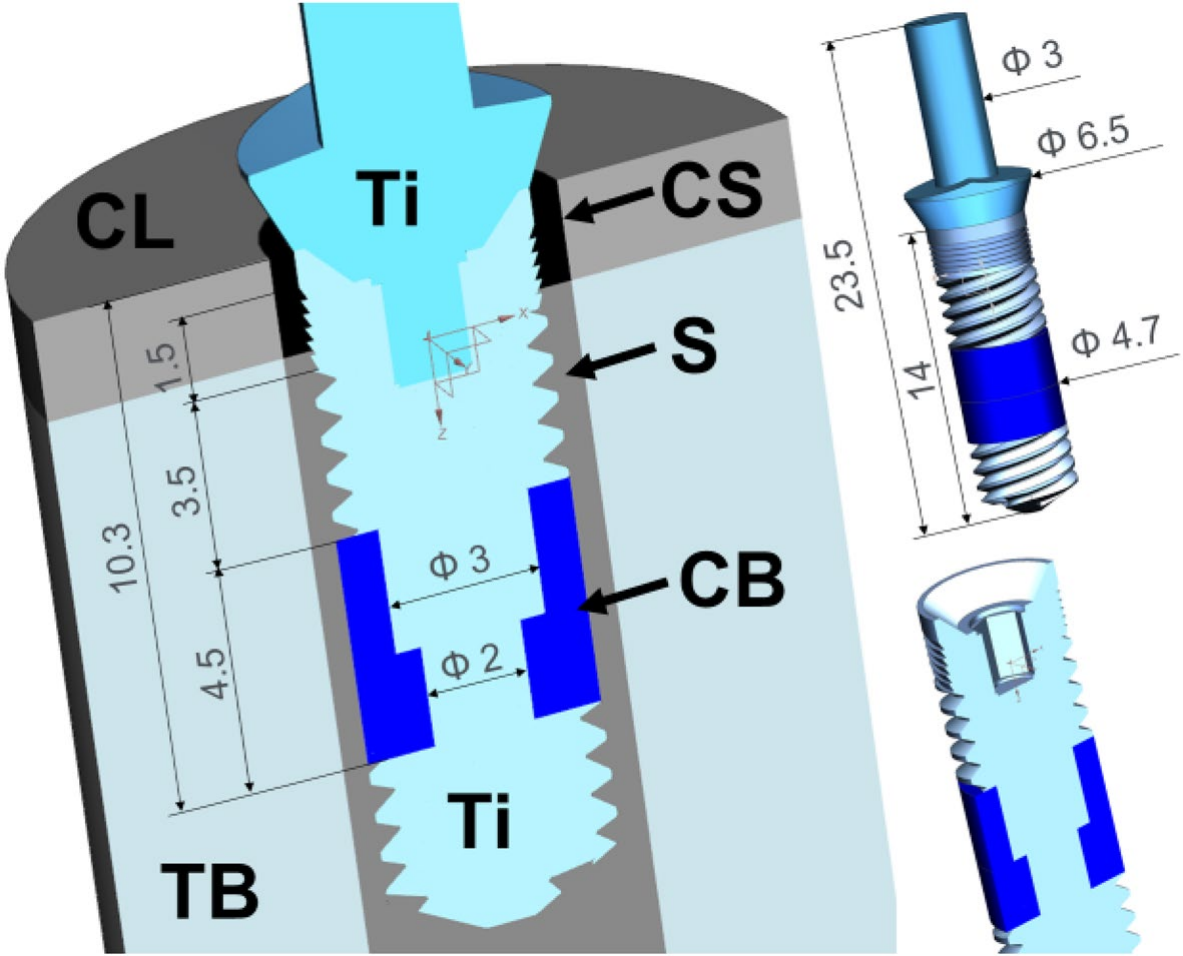
Compliant bushing (CB) around stepped titanium implant shaft and bones: *CL* cortical layer, *TB* trabecular bone, *CS* cervical support, *S* corticalized sheath.

Simulation tests were carried out for an implant with compliant bushings (CBs) in the middle part on a titanium stepped shaft. Figure 1 shows the implant model in section. The tests were carried out for CBs^23^ with an increasing modulus of elasticity of 2 GPa, 10 GPa and 50 GPa. Young’s modulus of porous tantalum can be tailored between 1.5 and 20 GPa by changing the pore volume fraction between 27 and 55%^3^. The material with the modulus of 50 GPa was used as a control towards bulk low elasticity Ti alloy^45^, although for porous Ta it reaches 30 GPa^6^. Implant shaft was made from pure titanium with the modulus of elasticity of 105 GPa^46,47^. Implant model was introduced into the cylindrical bone model with a 2 mm thick cortical bone layer. The value of 2 mm was taken as representative for an average cortical bone thickness, as it ranges from 1.09 to 2.12 mm in the maxilla and from 1.59 to 3.03 mm in the mandible^48^. Bone thickness affects the load distribution—a thicker layer of cortical bone is stiffer and bears a relatively larger part of the loads^49^. Cortical and cancellous bone tissues were isotropic linear elastic with the modulus of 15 GPa and 0.5 GPa^23,50,51^, respectively.

Various stages after implantation have been studied: primary stability and secondary stability after osseointegration^52–55^. For this purpose the bone sheath (S) around the implant was modeled with a 0.5 mm thickness^55,56^. In primary stability bone sheath (S) had the elastic modulus like the cancellous bone. A distinction between low and high quality cervical support for primary stability was investigated. The implant with low quality cervical support was simulated using a lower modulus of elasticity (0.5 GPa) for bone sheath (CS) of a 0.5 mm thickness around the implant neck (Fig. 1). Secondary stability conditions (the time of bone remodeling and densification may range from 6 to 18 months^57–59^ were simulated with stiffer woven corticalized bone sheath (CS and S with E = 15 GPa). The assumed distance and elasticity of corticalized bone were hypothesized on the basis of general views on corticalization around implants and the extent of the zone was adopted on the basis of illustrative cross-sections of implants after osseointegration^54^ and studies showing an increase in density at the assumed distance of 0.5 mm from the implant surface^56,60–62^. In addition, in order to obtain satisfactory accuracy, a division into 3 finite elements at the thickness of the intermediate zone was adopted. In the case of assuming a zone of 100–200 microns, it was not possible for us to count the model of the entire implant due to the size of the analysis.

The analysis of the oblique occlusal load transfer (250 N; 45°) was made using the FEA (SIMCENTER 3D, SIEMENS). The position of the implant in the dental arch was not given in the simulation, as the study was aimed at a relative comparison of the load distribution mechanism depending on the stiffness of the CB. The position of the implant should be considered in the context of assumed loads whose value and direction correspond to the range of average cyclic masticatory loads on molars or upper values for occlusion on incisors^43,63–73^. In addition, the linear model allows for proportional scaling of the results, which has also been described in the discussion in the context of the impact of their biomechanics on implant loadings.

Viscoelastic bone response was also omitted due to the cost of the analysis. The approach to bone as an elastic material and the aim of the work, which is the analysis of the load distribution mechanism, entitles us to simplify the analysis to a single static maximum load in a cycle, which by default is equal to the chewing cycle, i.e. about 1 Hz. The strength of the implant material in this range does not depend on the frequency but on the number of cycles, and we have simplified the bone to a linear elastic one. The statically calculated stress represents the situation for the maximum value in the unidirectional bending, i.e. assuming dominance in occlusal load cycles only in one direction outside the dental arch. However, this is only an assumption because occlusal forces can also act on the other side of the dental arch, leading to reverse bending and lower fatigue strength^74^.

In each test, the same finite element mesh was used, the convergence of which was achieved during preliminary tests. The increase in mesh density concerned the bone sheath area surrounding the implant and CBs. The nodal and elemental stress value (Fig. 3c,d) in the region of interest of corticalized bone sheet around the CB differed the most at the boundary with the trabecular bone, locally in single finite elements with the value between 1 and 2 MPa, which is a satisfactory value of 7–13% compared to the nominal stress value of 13–15 MPa. The computationally expensive special stress recovery procedure to achieve an exact solution for discontinuous gradient fields at the material interfaces^75,76^ was not used due to the achievement of the purpose of the work.

A simplification of bonded contact^21,24^ was assumed between all parts because of a significant cost increase of non-linear analysis with sliding contact.

### Ethical approval

This article does not contain any studies with human participants or animals performed by any of the authors.

## Results

**Figure 2:**
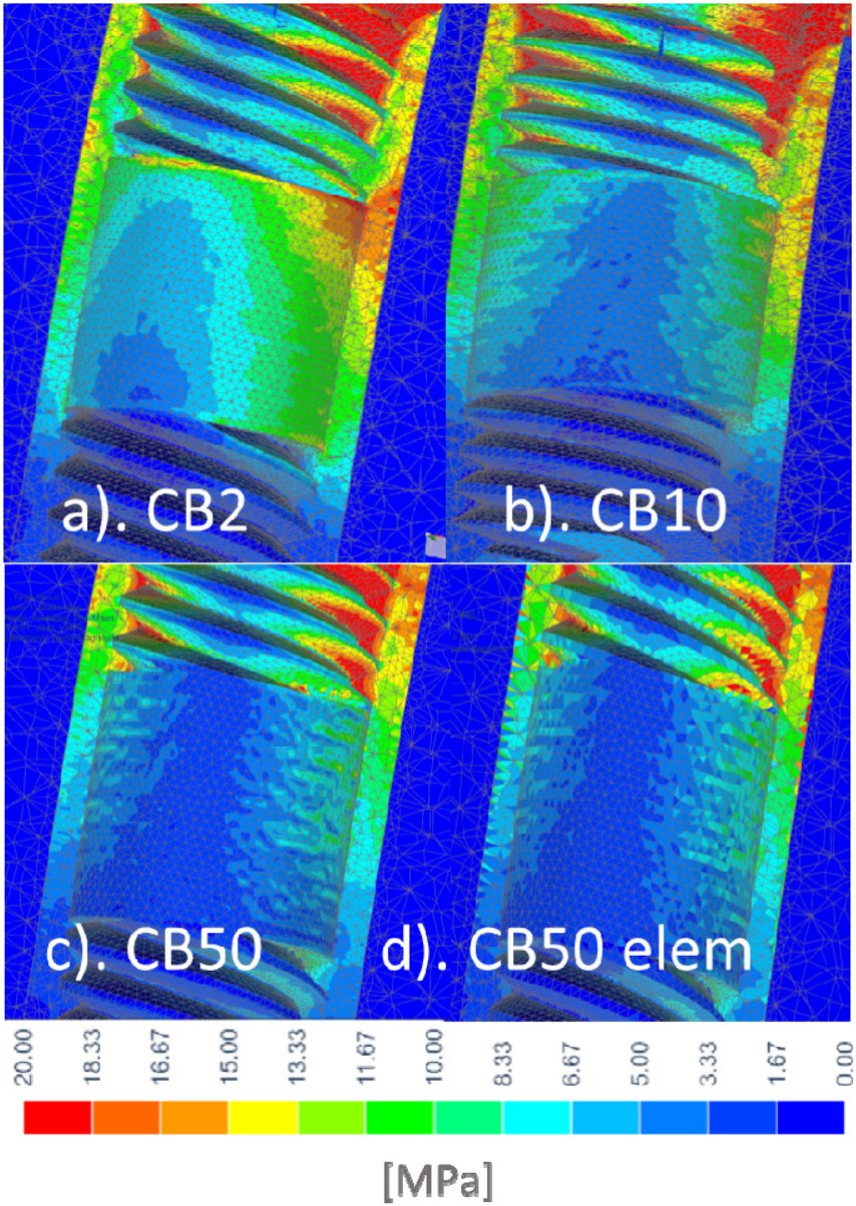
Influence of CB: (**a**) CB2, (**b**) CB10, (**c**) CB50 on equivalent HM stress in the corticalized bone sheath (E = 15 GPa) in secondary stability. Mesh size analysis showed satisfactory convergence of nodal values (**c**) with elemental values (**d**).

The values of equivalent stresses according to the theory of maximum distortional strain energy (Huber-Mises) were the criterion for assessing the impact of the CB on bone. The stresses in Fig. 2 relate to the case after osseointegration. As the porosity increased, the beneficial effect of a smaller Young's modulus of the porous bushings on stress in the surrounding bone was found. A beneficial increase in stress was seen along with a decrease in the modulus of elasticity of CB.

The more flexible CB bore less of the load and gave part of it to the surrounding woven mature bone sheath. As a result, the implant bent more in the bone in this area. Due to this, a much larger area of bone tissue begins to be physiologically active and stimulated to grow.

**Figure 3:**
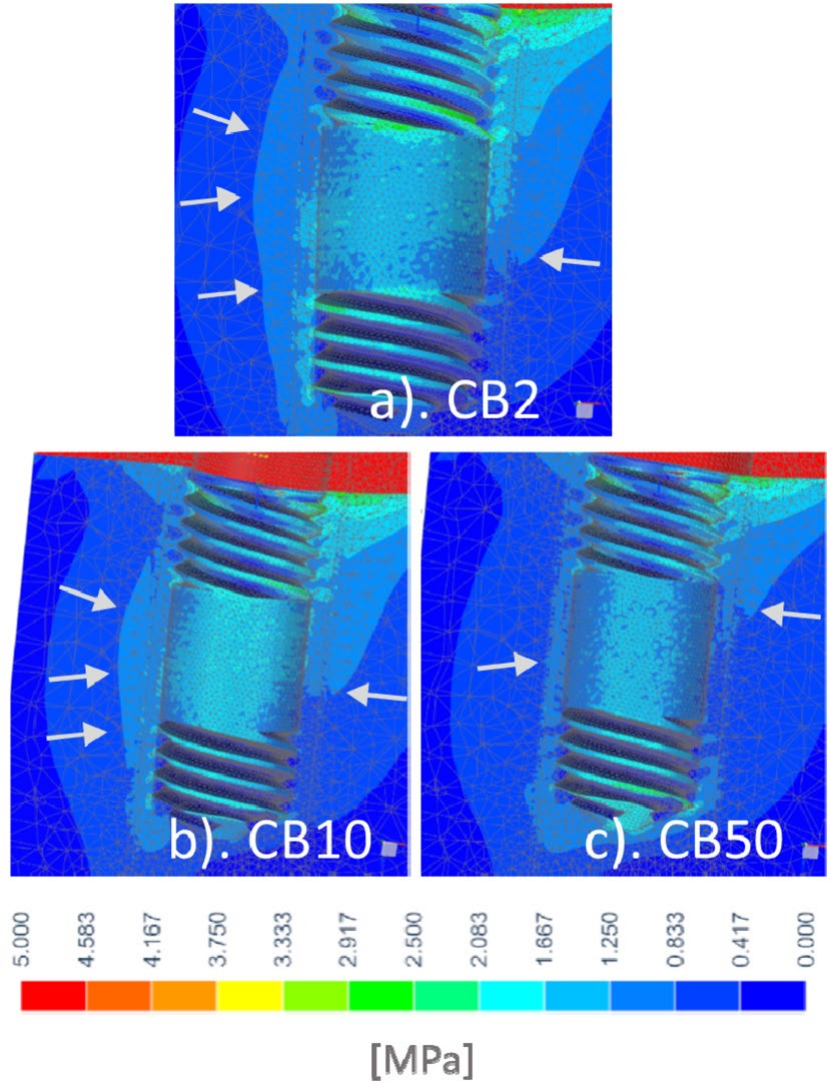
Influence of CB: (**a**) CB2, (**b**) CB10, (**c**) CB50 on equivalent stress in trabecular bone tissue for a model with high quality cervical support during primary stability.

**Figure 4:**
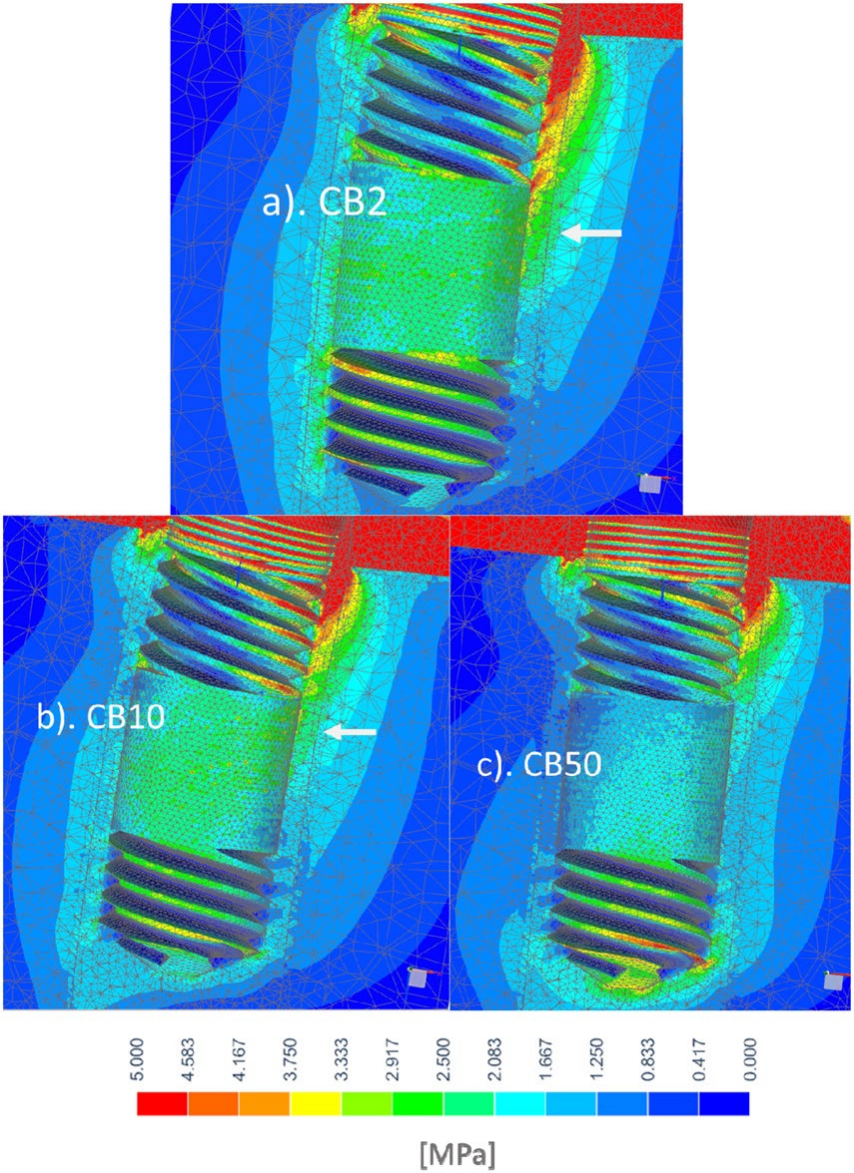
Influence of CB: (**a**) CB2, (**b**) CB10, (**c**) CB50 on equivalent stress in trabecular bone tissue for the model with low quality cervical support during primary stability).

Stresses in the case of low and high quality cervical support during primary stability were shown on Figs. 3 and 4, respectively. Also in the cancellous bone before the creation of woven mature bone, with a decrease in the modulus of elasticity of CB, a much larger area of bone tissue works more favorably. At the same time, the implant tip moved less as a result of greater deflection in the bushings. There was a favorable equalization of stress along the endosteal part of the implant.

**Figure 5:**
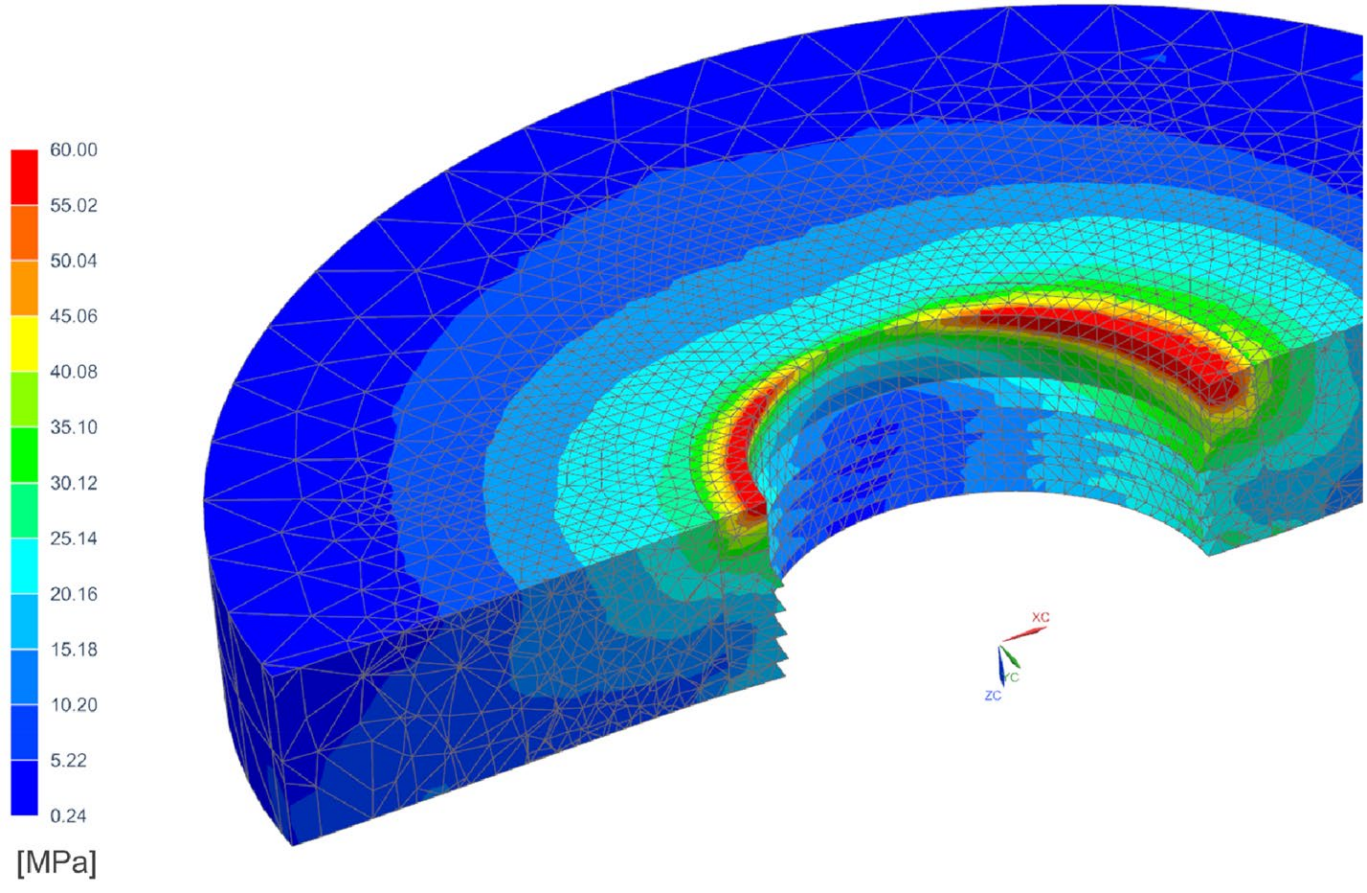
Equivalent stress in cortical bone tissue in a model with secondary stability after osseointegration for CB50.

Figure 5 shows the stress distribution around the implant neck in secondary stability in the case of CB50. In the area closest to the margin, the stress reached a dangerous level for atrophy, which is consistent with the results of other FEA occlusal load distribution and the clinical funnel-like atrophy around the implant neck^76–82^. Stresses in the cortical bone around the implant neck were similar for various CBs, therefore the impact of CBs concerned deeper endosteal zones where greater implant flexibility is crucial for stress redistribution.

**Figure 6:**
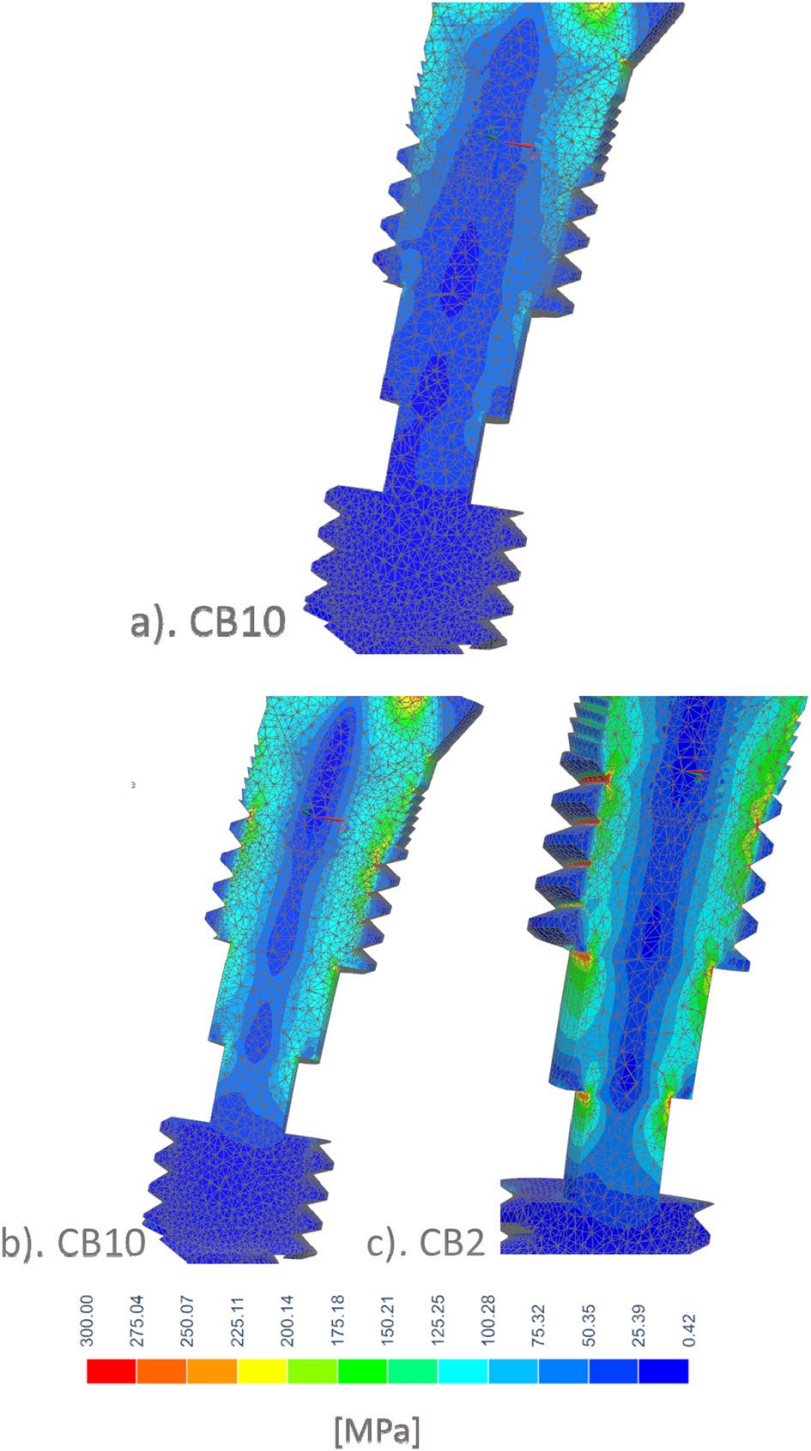
The effect of CB on equivalent stress in a titanium shaft in the case of (**a**) CB10 high quality cervical support. (**b**) CB10 and (**c**) CB2 low quality cervical support (CS).

Stresses in titanium shaft increased with increasing porosity of CBs to a small extent. Low quality cervical support increased the bending stress much more than CB elasticity, which is presented in Fig. 6. The stress was at a safe level for pure titanium^83,84^, especially considering stress overestimation near sharp edges, where stress of about 300 MPa is only an artifact of FEM and true values are lower^76^.

**Figure 7:**
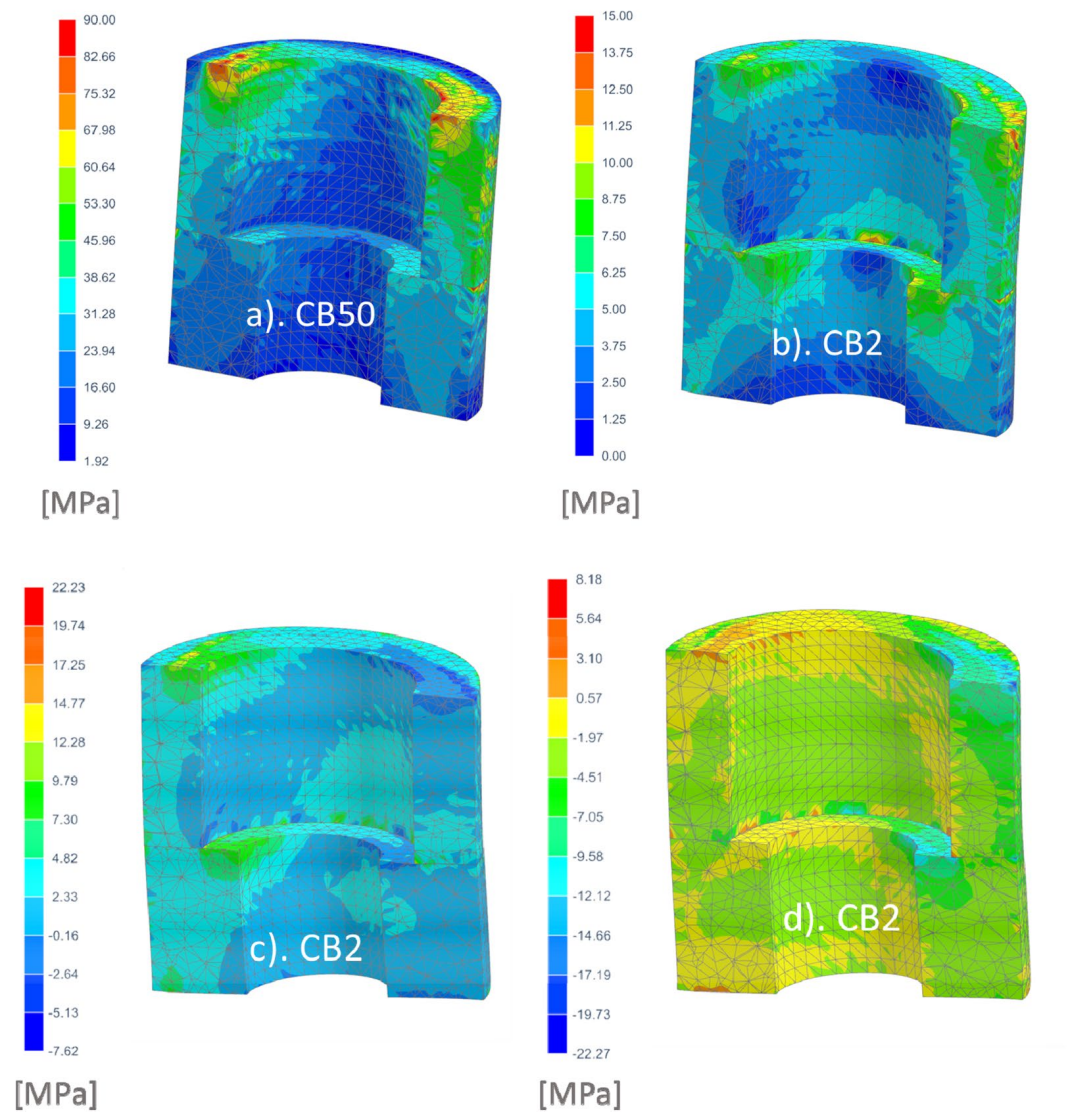
Stress in the CB in an implant with low quality cervical support: (**a**) CB50 equivalent H-M. (**b**) CB2 equivalent H-M. (**c**) CB2 maximal principal. (**d**) CB2 minimal principal.

Stress in the CBs decreased with an increase in their compliance (Fig. 7). Stresses were at a safe level for PTs produced by different technologies^8,23^. PTs manufactured by CVD/CVI with the modulus of elasticity of 2.5–3.9 GPa show ultimate compressive strength in the range of 50–70 MPa and yield the strength of 35–51 MPa and tensile strength of 63 MPa^23,34^. PTs made by powder metallurgy have the modulus of elasticity of 2.05–2.37 GPa.

GPa shows the compressive strength of 57–66 MPa^85^. The PT produced by replication of NaCl space-holders^9^ with the modulus of 1.7–2.3 GPa exhibits the compressive strength of 48.8–51.8 MPa. The PT manufactured by CVD / CVI is still better tested in terms of mechanical properties and clinically proven. Compressive fatigue strength of PT for 5 × 10^6^ cycles reaches the value of 23 MPa and 35 MPa for cantilever bending^8^. Structure investigations show failure on the tension side. On the other hand, there is a significant statistical spreading of compressive strength^8^. The PT with the modulus value of 1.3 ± 0.6 GPa shows the static compressive strength of 55 ± 38 MPa^8^. During compressive fatigue some samples were damaged for values below 15 MPa. The results of simulation studies indicate that minimal principal stress does not reach such values, because after separating FEM artifacts they reached about 10 MPa. However, it should be noted that critical areas are compressed and tensioned circumferentially. It should also be remembered that the bite force often changes direction and a number of cycles cause bending in the opposite direction. Fatigue strength of PT in such loadings is unknown.

**Figure 8:**
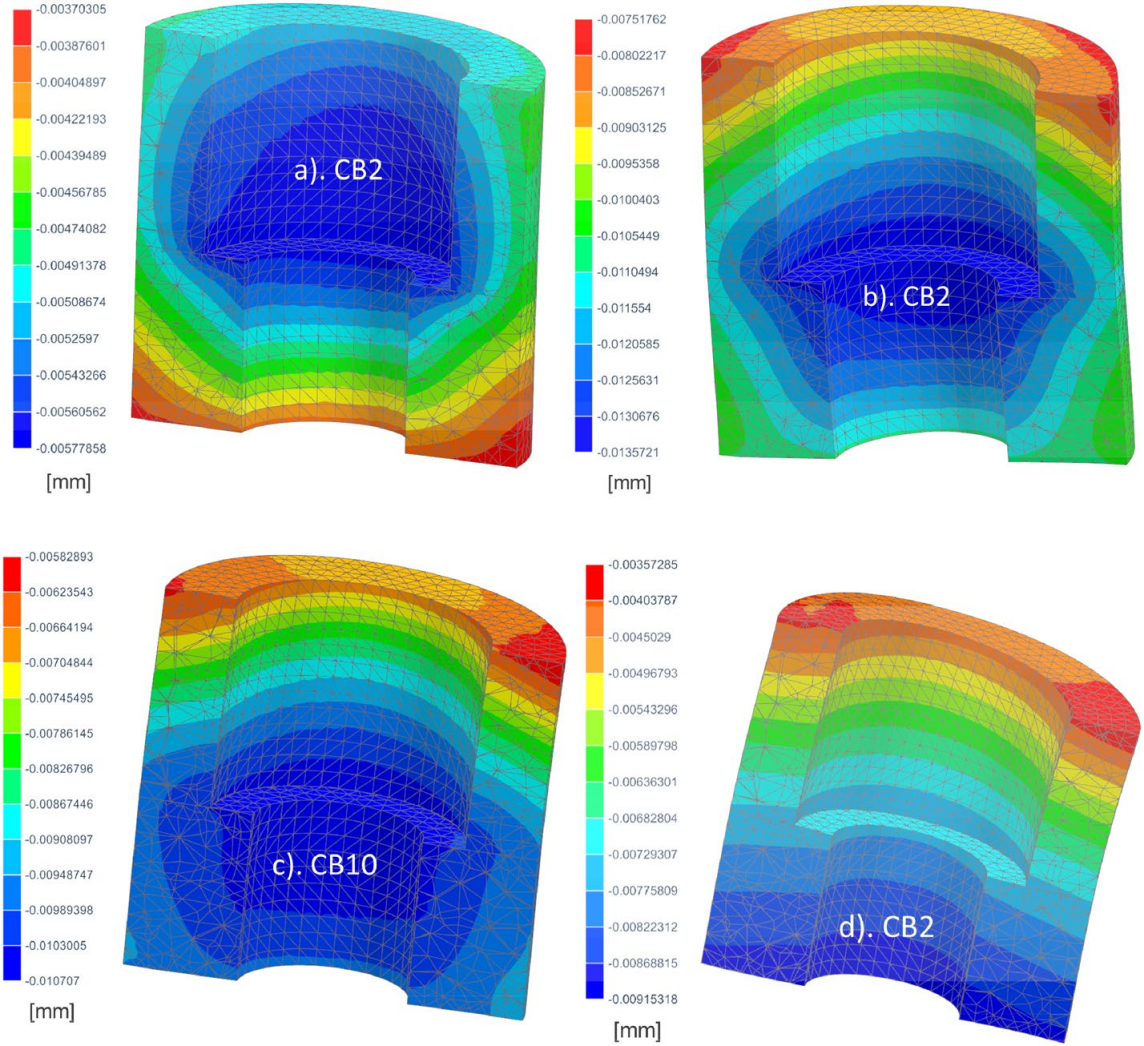
Displacement in X direction in CBs: (**a**) CB2- high quality cervical support (**b**) CB2-low quality cervical support (**c**) CB10 low quality cervical support (**d**) CB50 low quality cervical support.

Transverse deformations in Fig. 8 (in X direction along horizontal component of bite load) explain the mechanical behavior of CBs. The bone implant experiences horizontal displacements in the opposite direction to the horizontal bite force component. As the compliance of CBs increases, the nature of the displacement field changes significantly. Stiffer CBs (CB50) in the case of the implant with low quality cervical support had the largest displacements at the bottom. In the less rigid CBs (CB10) the largest displacements move up. In the most susceptible case (CB2-low qualityCS) they move to the central area and in the case of high quality support they move even higher to the area of higher bushing.

## Discussion

The development of technologies that allow pore wall thickness control has increased the possibility of clinical use for load-bearing orthopedics implants. However, for porous structures of 100–200 microns, their wall thickness is so small that the deviations of additive technology based on metal powders and melting/printing are significant due to the powder size and melting zone. This problem has been eliminated in the chemical vapor deposition/infiltration technology (CVD/CVI) of tantalum on the carbon framework, which is prepared as a result of pyrolysis of polymer foam^8,23,25^. PT made with the vapor deposition technology has shown good corrosion–erosion resistance in comparison to titanium and stainless steel implants^86^.

Dental implants with a PT bushing after 12 weeks in rabbit tibiae undergo osseointegration and show the stiffness of the bone-implant interface similar to threaded titanium implants^87^. However, in dental implantology, long-term mechanical complications often occur, when bone atrophy results in the implant being exposed from the bone after several years as a result of stress shielding and micro-leakages into gaps under dental abutments. The porosity of dental implants in the cervical zone in the case of marginal bone loss increases the risk of peri-implantitis^88^ and further atrophy, which, together with the increase in the leverage of lateral forces, increases the risk of fracture^89^. Currently, however, there is no long-term clinical data on whether marginal bone loss may occur to the depth of PT, meanwhile there are promising data that PT may slow down marginal bone loss^30,90^. Meanwhile, studies^87^ concerning the initial period and resonance frequency analysis were used to assess stiffness, which does not provide information about the state of stress around the porous zone. A wider knowledge was provided by clinical work^30^, in which 64% better bone condition around PT implants was found compared to standard titanium implants.

In the cervical region bone atrophy at the level of a few millimeters after several years is classified as a clinical success^76–82^. The formation of a stiffer sheath around the endosteal part provides secondary implant stability. Stresses in the stiffer bone sheath in the case of a reduction in the modulus of elasticity of the CB to 2 GPa increased around the bushings to a range that better activates bone tissue growth. In the zone of the neutral bending plane, stresses increased from about 3 Mpa to over 6 Mpa. In the case of immediate loading and high quality support similar effects are visible. It is worth noting the effect of the shift of stresses in the upper part from higher to deeper threads. This effect is also visible in the case of low quality support, especially on the tensioning side. The results of simulation studies are consistent with the clinical data of the works^25,30,87,90,91^ where the porous zone increases static and dynamic stiffness values and the implant-bone contact when compared with standard threaded implants.

Our research, although performed for the same implants, is not comparable with the results of the study^44^ in which the force values for the subsequent implants are not known, because they work under implant-retained denture. The loads on the implants under implant-retained denture depend on the stiffness of the implant in the bone and the denture attachment^92,93^. Porous implants, due to their lower rigidity in the bone, distribute occlusal loads differently, therefore a comparison when working with a denture is not reliable.

Similar tendencies are visible in the work^20^, where for implants with a porous radial zone, in the case of a decrease in the modulus of elasticity in the outer zone in a similar range from 53.84 to 9.11 Gpa, the eqvH-M stress increases from 2 to 2.5 Mpa. However, in this work trabecular bone with a much higher modulus of elasticity, axially oriented implant load and implant designs are different, so the results cannot be directly compared. Similar results were obtained in the work^65^, in which stresses of 2.5 Mpa are evenly and widely distributed in the trabecular bone around a similar implant with a porous medial part under an oblique load of 100 N (45°), but the lack of bone modulus does not allow for further attempts to compare the results. Also, a direct comparison with the results from the photoelastographic study^94^ is impossible due to the different loading and supporting conditions of the model, but there is a similar trend of increasing stress in the bone tissue around the implant with increasing porosity and compliance in the middle zone. Similar trends are visible at work^95^ where under oblique loading of 118.2 N there is an increase from 1 to 9% of the proportion of cancellous bone around the implant that has the strain in the range of 1500–3000 με with the porosity from 34.08 to 74.5% of the implant shaft. Also in the work^24^ stresses in cancellous bone are more evenly distributed as a result of increasing the use of cancellous bone support when pressing more flexible implants into the bone, in which the metallic titanium core was surrounded by flexible PEEK.

In our studies, the increase in stress was seen for all cases of bone conditions. The case of perfect corticalization was purely hypothetical. The thickness of corticalized zone was adopted on the basis of illustrative cross-sections included in the work^54^. However, a more detailed insight into the current data will not allow for unambiguous confirmation of the assumed thickness and elasticity of this zone. There are considerable discrepancies regarding the extent of the zone and the properties of corticalized bone tissue around implants, however, the density around functionally loaded dental implants increases even if marginal bone loss occurs^61,62,96–98^.

The works^55,56^ documented the increasing density of peri-implant bone at distances 0.5 mm and 1.0 mm from lateral threaded surface, however the elasticity of newly formed bones is unknown. Elastic modulus of newly formed bone tissue surrounding an implant measured with nanoindentation in a rabbit tibiae model^52^ ranges from 15.35 to 17.82 Gpa during a 4–13 week healing period and is not far away from the value of 20.66 Gpa achieved for mature bone, however it concerns bone tissue formed in a gap between the implant and cortical bed.

We note that the research was focused on the beneficial effect of increasing stress, which helps to protect the atrophy of cancellous bone tissue from disuse. The research was therefore one-sided. Full evaluation requires the use of a two-sided optimization criterion including the overload criterion. In the Frost’s criterion according to the mechanostat theory the range of safe strain is < 3000 µε^99,100^ what corresponds to stress below 1.5 Mpa for trabecular bone with a modulus of 500 Mpa.

In our studies, in the case of low quality cervical support during primary stability in the vicinity of the upper part of the CB2 and even CB10, strains of about 6000 µε (3 Mpa) occurred, and locally slightly higher values (Fig. 4), which resulted in bone damage from cyclic overloading. The proportionality for the linear model shows that in the case of low quality primary stability it is possible to transfer cyclic loads below half of the applied force of 250 N. Nevertheless, at 250 N the stress was below bone fracture risk (< 10,000–25,000 µε, < 5 ÷ 12.5 Mpa), albeit it should be remembered that during primary stability a simplified bonded contact was assumed, while the actual conditions are then less favorable due to the lack of osseintegration. It is also evident that the critical area that occurs at the cortical bone unfortunately widens significantly as the stiffness of the implant decreases.

The stress in the critical area in the case of high quality of primary stability also reached dangerous values for bone loss and was enlarged with bushing compliance, but around bushing was only locally at the upper edge at thread (Fig. 4). In the case of hypothetical corticalization with an elasticity of 15 Gpa, strains exceeded the threshold of bone homeostasis only for B2 (> 1000 µε, > 15 Mpa). Under the conditions of the model, it would therefore be possible to maintain bone of such high quality, but not gain around CB2.

The results confirm that the placement of this type of implant in the lateral zones requires further research in the conditions of primary stability with contact phenomena to assess the increase in the risk of bone loss from overload under the full range of masticatory forces. The results indicate that obtaining the benefits of CB requires high quality of stability or load reduction.

The results confirm the opinion that a safer place for such implants are the anterior zones or under implant-retained soft-tissue supported dentures. In the case of two-implant retained dentures oblique mastication force of 141 N (45°) produces a lateral force of about 40 N and an axial force of 60 N on the implant^93^. Hence the implant oblique loads of 72.1 N are about 3.5 times smaller and at a gentler angle than the assumed 250 N in the analysis. In the case of a single implant-retained denture, the implant bears a lateral force of 52.5–84 N and is pulled out of the bone with a force of 72.6 N^92^, which, ignoring the direction of axial loads, results in slightly more than two times smaller load.

The model has some limitations. No contact phenomena have been modeled on the bone surface, which reduce the tensile values on the tensioning side and increase compressive values on the compression side. Viscoelastic response of bone and anisotrophy are other omitted parameters. Deformation of the whole bone is omitted which can influence local stress during mandible bending. In regard of the requirement to adjust the cost of calculations to the available computer (i5-7400 3 GHz, RAM 8 GB), no further mesh enhancement and coincidence between bodies were carried out. Stress field can be tuned with a better mesh density and more accurate values can be obtained at the material interfaces by applying the stress recovery procedure for discontinuous gradient fields.

## Conclusions

Reducing the stiffness of the bushings surrounding the stepped implant shaft increased stress in the trabecular bone, protecting it from atrophy from disuse for a model with high quality cervical support during primary stability, however, in the case of low quality cervical support, the stress reached dangerous values in terms of the risk of overload atrophy, although the stress in the implant shaft was below fatigue strength.

The obtained results confirm the opinion that it is safe to place such implants in the anterior area of the dental arch or under implant-retained tissue supported dentures, however, further research with regard to contact phenomena is needed to assess the risk of atrophy due to overload for various bone conditions.

## Data Availability

The datasets used and/or analysed during the current study are available from the corresponding author on reasonable request.
